# Clinical characteristics and caloric testing in patients with light or heavy cupula of the horizontal semicircular canal

**DOI:** 10.3389/fneur.2025.1620972

**Published:** 2025-08-01

**Authors:** Xueyan Zhang, Jiao Xu, Tao Zhou, Xue Yu, Jin Xu, Heng Yu, Guangjie Song, Lingli Wei, Xu Yang, Mei Hu, Liying Chang

**Affiliations:** ^1^Department of Neurology, Xiangyang Central Hospital, Affiliated Hospital of Hubei University of Arts and Science, Xiangyang, China; ^2^Department of Neurology, Peking University First Hospital, Beijing, China

**Keywords:** light cupula, heavy cupula, sudden sensorineural hearing loss, null point, canal paresis

## Abstract

**Objectives:**

Persistent direction-changing positional nystagmus (DCPN) and null point (NP) are characteristic of cupulopathy of the horizontal semicircular canal (HSC). The cupulopathy can manifest as HSC-light cupula (HSC-Lcu) (geotropic DCPN) and HSC-heavy cupula (HSC-Hcu) (apogeotropic DCPN) in the supine roll test (SRT). Whether the affected side of cupulopathy could be based on the nystagmus intensity in the SRT is controversial. This study aims to explore the differences in clinical characteristics and the HSC function between the HSC-Lcu and HSC-Hcu.

**Methods:**

In this retrospective study, the disease histories of patients were collected, including those of peripheral vestibular disorders, otological diseases, and neurological diseases. We compared the nystagmus characteristics and canal paresis (CP) between the two groups. A multivariable logistic regression analysis was performed to identify predictors of cupulopathy subtype classification.

**Results:**

We included 52 patients with HSC-Lcu (17 males; mean age: 66.6 years) and 47 patients with HSC-Hcu (24 males; mean age: 68.0 years). A history of sudden sensorineural hearing loss (SSNHL) was more common in patients with HSC-Lcu (*n* = 8) than in those with HSC-Hcu (*n* = 0) (*p* = 0.005). There was no significant difference in NP1, NP2, or NP3 between the groups. The NP2 is present in all patients with HSC-Hcu. The side with stronger nystagmus intensity during the supine roll test (SRT) was consistent with the NP side in 38 patients with HSC-Lcu and 21 patients with HSC-Hcu. CP was more frequent in patients with HSC-Lcu (*n* = 29) than in those with HSC-Hcu (*n* = 16) (*p* = 0.030). However, when evaluated within a multivariable logistic regression model, the presence of CP was not found to be statistically significantly associated with the outcome (*p* > 0.05).

**Conclusion:**

A history of SSNHL specifically associates with HSC-Lcu, rather than HSC-Hcu. Determining the affected side of HSC-Lcu and HSC-Hcu based on nystagmus intensity during the SRT was unreliable. HSC-Lcu shows higher rates of CP, indicating that the function of HSC-Lcu was more impaired than that of HSC-Hcu. Clinicians should consider SSNHL history and perform caloric testing when evaluating suspected HSC-Lcu, and rely on the NP for lateralization.

## 1 Introduction

Vestibular disorders affecting the semicircular canals represent a significant global health burden, contributing to disabling vertigo, imbalance, and increased fall risk (1, 2). Accurate localization of the affected side is needed for the effective management of peripheral vestibular pathologies. The horizontal semicircular canal (HSC) is particularly susceptible to conditions involving an abnormal cupular-to-endolymph specific gravity ratio: horizontal canal light cupula (HSC-Lcu) and horizontal canal heavy cupula (HSC-Hcu). When cupular specific gravity is lower than that of endolymph (light cupula), the cupula deflects in the utriculofugal direction during the supine roll test (SRT), resulting in persistent geotropic direction-changing positional nystagmus (DCPN) (lasting >1 min) according to Ewald’s law (3). Conversely, when cupular specific gravity is higher than that of endolymph (heavy cupula), the cupula deflects in the utriculopetal direction, generating persistent apogeotropic DCPN (lasting >1 min) (4, 5).

Clinical observations indicate that cupulopathy is frequently associated with a history of peripheral vestibular disease, including BPPV, vestibular neuritis, sudden sensorineural hearing loss (SSNHL), otitis media, and neurological diseases, including vestibular migraine and stroke (6–10). However, the differences in disease histories of the two cupulopathy subtypes (HSC-Lcu and HSC-Hcu) remain unclear. It is deserved to comprehend these relationships to elucidate the pathogenesis, given that different etiological factors may influence otoconial density and cupular adhesion mechanisms. For instance, inner ear disorders such as benign paroxysmal positional vertigo (BPPV) have been demonstrated to disrupt otolith membrane integrity (11).

The sensory transduction mechanism within the crista ampullaris relies on cupular deflection caused by endolymph flow during head movement. The null point (NP), also known as “null plane” or “zero plane,” which is defined as a specific head orientation that restores cupular-endolymph equilibrium and ceases nystagmus, is a characteristic of both HSC-Lcu and HSC-Hcu (12).

The affected side in horizontal semicircular canal-light cupula (HSC-Lcu) and horizontal semicircular canal-heavy cupula (HSC-Hcu) can be determined by identifying the side of the NP (13–16). Previous studies have proposed that the affected side in HSC-Lcu and HSC-Hcu could be identified based on nystagmus intensity observed during the SRT. In light cupula, the affected side is considered ipsilateral to the stronger nystagmus intensity, whereas in heavy cupula, it is considered contralateral to the stronger intensity. However, this method remains controversial (13, 17, 18). Our study aimed to explore whether nystagmus intensity in the SRT could be used to determine the affected side of cupulopathy.

The caloric test is a widely used method to assess vestibular function through bithermal irrigation with water or air (19–21). Caloric testing has been used to assess HSC dysfunction in patients with HSC-Lcu and HSC-Hcu (10, 22). However, there is a lack of comparative data on the prevalence and lateralising value of CP between HSC-Lcu and HSC-Hcu patients.

## 2 Methods

This retrospective study was conducted at the Department of Neurology, Xiangyang Central Hospital, the Affiliated Hospital of Hubei University of Arts and Science. The source population comprised adult inpatients who were evaluated and treated for vertigo/dizziness within the Department of Neurology between November 2020 and July 2023. Study data were extracted exclusively from the comprehensive electronic Hospital Information System (HIS).

### 2.1 Inclusion and exclusion criteria

Patients exhibiting persistent geotropic or apogeotropic DCPN (lasting >1 min) without latency or fatigability in the SRT were included. The following patients were excluded: (1) Intracranial lesions confirmed by magnetic resonance imaging (MRI). (2) Presence of other types of nystagmus identified during the Dix-Hallpike test (D-HT). (3) Evidence of central vestibular or neurological lesions detected through detailed neurological and neurotological examinations. (4) Failure of visual fixation to suppress caloric-induced nystagmus (indicative of central pathology).

### 2.2 Study design and sample size

This was a single-center, retrospective cohort study conducted using data extracted from the HIS. All inpatient records within the Neurology Department during the study period (November 2020 to July 2023) were screened for eligibility based on the documented presence of persistent geotropic or apogeotropic DCPN during SRT testing within the HIS. As this was a retrospective study encompassing all eligible cases within the specified timeframe, a formal prospective sample size calculation was not performed. The analysis included all patients who met the predefined inclusion and exclusion criteria during the study period. A consecutive sampling approach was utilized. We finally obtained a total of 107 eligible patients (55 patients with HSC-Lcu and 52 patients with HSC-Hcu) meeting the criteria during the study period.

### 2.3 Data collection tools and procedures

Demographic information, medical history, including history of peripheral vestibular disorders and otological disease [benign paroxysmal positional vertigo (BPPV), vestibular neuritis, Meniere’s disease, otitis media, and sudden sensorineural hearing loss (SSNHL)] and history of neurological disease (stroke and vestibular migraine), and clinical examination findings were systematically extracted from the HIS.

In the D-HT, the patient sat on the examination table with the head turned 45° to one side and eyes kept open, then was quickly moved to a position with the head slightly hanging (20–30°) over the edge of the table. Nystagmus was recorded in this position. After nystagmus remission, the patient returned to the sitting position, and the same procedure was performed on the opposite side.

In the SRT, the patient lay on the examination table with the head tilted forward 30° initially. After nystagmus subsided, the head was turned 90° to one side. Once nystagmus resolved, the patient returned to the supine position, and the same procedure was repeated on the opposite side. Nystagmus was recorded using a videonystagmography (VNG) system, VertiGoggles (ZT-VNG-II, Shanghai ZEHNIT Medical Technology Co., Ltd., Shanghai, China). The maximum slow-phase velocity (SPV) of the nystagmus within 10 s was automatically recorded by the VNG system.

The null point (NP)—NP1, NP2, and NP3—was identified as follows: NP1, when spontaneous nystagmus disappeared as the head was bent forward or backward in the pitch plane; NP2, when nystagmus disappeared as the head was turned to the left or right in the supine position with the head tilted forward 30°; NP3, when nystagmus disappeared as the head was turned to the left or right with the head bent forward 90° (9). Based on previous studies, the affected sides in HSC-Lcu and HSC-Hcu were determined to be ipsilateral to the side of the NP (13–16).

The caloric test was performed in the supine position, using bithermal (30°C and 44°C) caloric irrigation with water. Cold water was first irrigated into the right and left ears, followed by warm water into both ears. A 5-min interval was maintained between irrigations to avoid residual effects. Each irrigation lasted 30 s using 250 mL of water. The CP value was calculated using the Jongkees formula (23), and a CP value ≥ 25% was considered indicative of canal dysfunction.

### 2.4 Statistical analysis

All statistical analyses were conducted using SPSS (version 28.0, Chicago, IL, United States). Categorical data were expressed as frequencies and percentages, and comparisons were made using the chi-square test. The normality of continuous data was assessed using the Shapiro–Wilk test. Continuous data with a normal distribution were expressed as mean ± standard deviation (SD), and differences were analyzed using the independent-samples t-test.

A multivariable logistic regression analysis was performed to identify predictors of cupulopathy subtype classification (HSC-Lcu and HSC-Hcu). The model was adjusted for age (continuous), sex (binary), history of SSNHL (binary), history of stroke (binary), and CP values (continuous). Goodness-of-fit of the logistic regression models was assessed using the Hosmer-Lemeshow test. The results are reported as odds ratios (OR) with 95% confidence intervals. All tests were two-tailed, and *p* < 0.05 was considered statistically significant.

### 2.5 Ethical consideration

The Ethics Committee of Xiangyang Central Hospital, Affiliated Hospital of Hubei University of Arts and Science approved this study (2022-069). All patients provided written informed consent.

## 3 Results

### 3.1 Patient selection and exclusion

**Figure 1:**
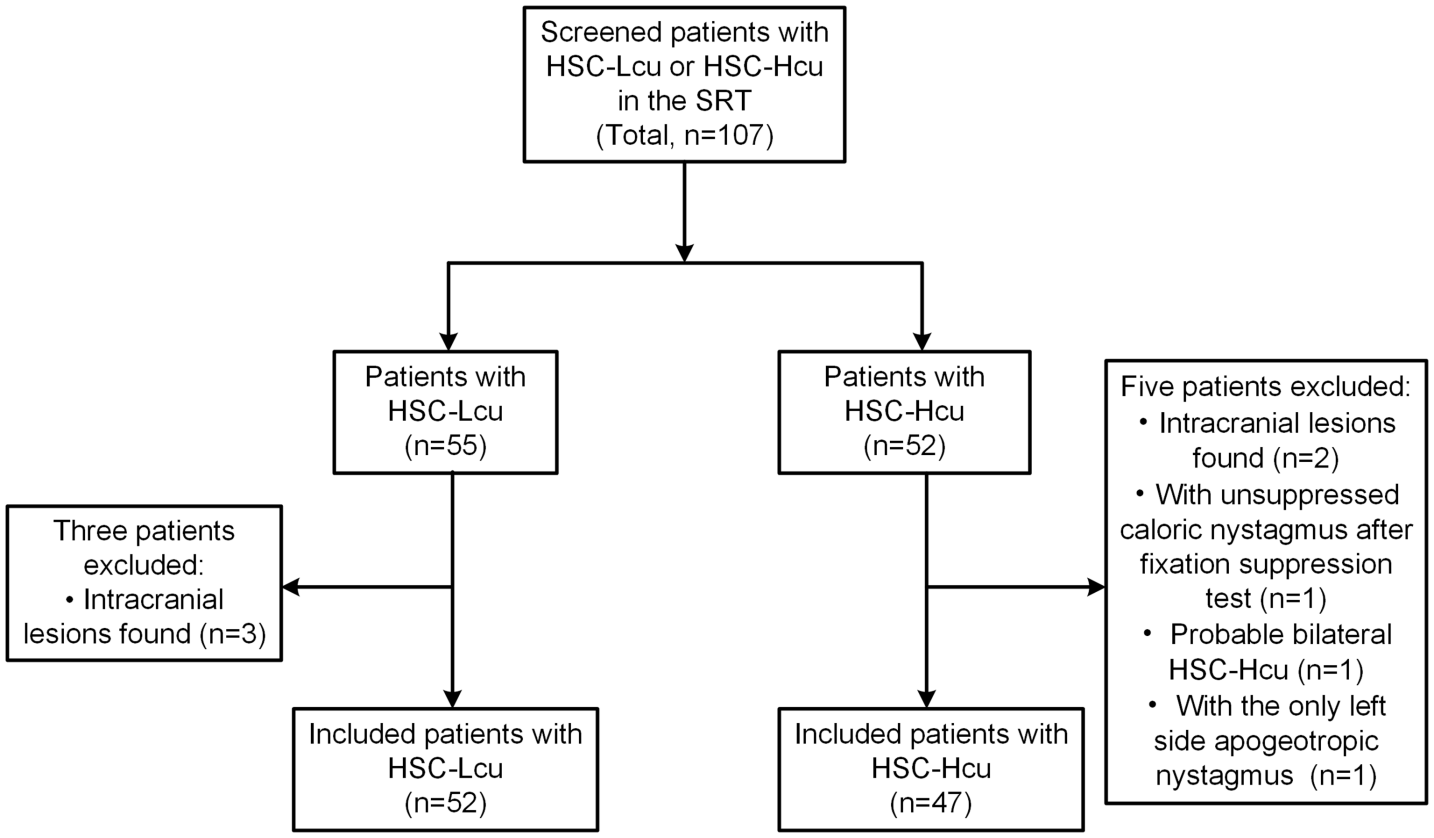
Flowchart of patients included. HSC-Lcu, horizontal semicircular canal light cupula; HSC-Hcu, horizontal semicircular canal heavy cupula; SRT, supine roll test.

We obtained 55 patients with HSC-Lcu and 52 patients with HSC-Hcu. We excluded three patients with intracranial lesions from the HSC-Lcu group. In the HSC-Hcu group, two patients with intracranial lesions, one patient with unsuppressed nystagmus after the visual fixation suppression test during the caloric test, one patient with probable bilateral HSC-Hcu (with bilateral NP2 and NP3), and one patient with only left-sided apogeotropic nystagmus were excluded. Finally, we included 52 patients with HSC-Lcu (17 males and 35 females; mean age ± SD = 66.6 ± 10.3 years; range: 43–98 years) and 47 patients with HSC-Hcu (24 males and 23 females; mean age ± SD = 68.0 ± 9.9 years; range: 42–90 years) (Figure 1).

### 3.2 Demographic and disease histories

There were no significant differences in age and sex between the two groups (*p* = 0.486 and *p* = 0.064, respectively). Regarding the history of peripheral vestibular disorders and otological diseases, patients with sudden sensorineural hearing loss (SSNHL) were more common in the HSC-Lcu group (*n* = 8) than in the HSC-Hcu group (*n* = 0) (*p* = 0.005). There were no significant differences between the two groups in the history of BPPV (HSC-Lcu: *n* = 1; HSC-Hcu: *n* = 4; *p* = 0.135), vestibular neuritis (HSC-Lcu: *n* = 5; HSC-Hcu: *n* = 3; *p* = 0.556), Meniere’s disease (HSC-Lcu: *n* = 2; HSC-Hcu: *n* = 1; *p* = 0.618), otitis media (HSC-Lcu: *n* = 2; HSC-Hcu: *n* = 1; *p* = 0.618), stroke (HSC-Lcu: *n* = 1; HSC-Hcu: *n* = 5; *p* = 0.007), or vestibular migraine (HSC-Lcu: *n* = 4; HSC-Hcu: *n* = 2; *p* = 0.474) (Table 1).

**Table 1:** Comparisons of demographics and disease histories between patients with HSC-Lcu and HSC-Hcu.

Demographics and disease histories	HSC-Lcu	HSC-Hcu	*p* value
	(*n* = 52)	(*n* = 47)	
Age, years			
Mean ± SD	66.6 ± 10.3	68.0 ± 9.9	0.486
Range	43–98	42–90	NA
Male, *n* (%)	17 (32.7)	24 (51.1)	0.064
History of peripheral vestibular disorders and otological disease, *n* (%)			
BPPV	1 (1.9)	4 (8.5)	0.135
Vestibular neuritis	5 (9.6)	3 (6.4)	0.556
Meniere’s disease	2 (3.8)	1 (2.1)	0.618
Otitis media	2 (3.8)	1 (2.1)	0.618
SSNHL	8 (15.4)	0 (0)	0.005^**^
History of neurological disease, *n* (%)			
Stroke	1 (1.9)	5 (10.6)	0.070
Vestibular migraine	4 (7.7)	2 (4.3)	0.474

^**^p < 0.01. BPPV, benign paroxysmal positional vertigo; HSC-Lcu, horizontal semicircular canal light cupula; HSC-Hcu, horizontal semicircular canal heavy cupula; *n*, number; NA, not applicable; SD, standard deviation; SSNHL, sudden sensorineural hearing loss.

### 3.3 Null points and nystagmus characteristics

**Figure 2:**
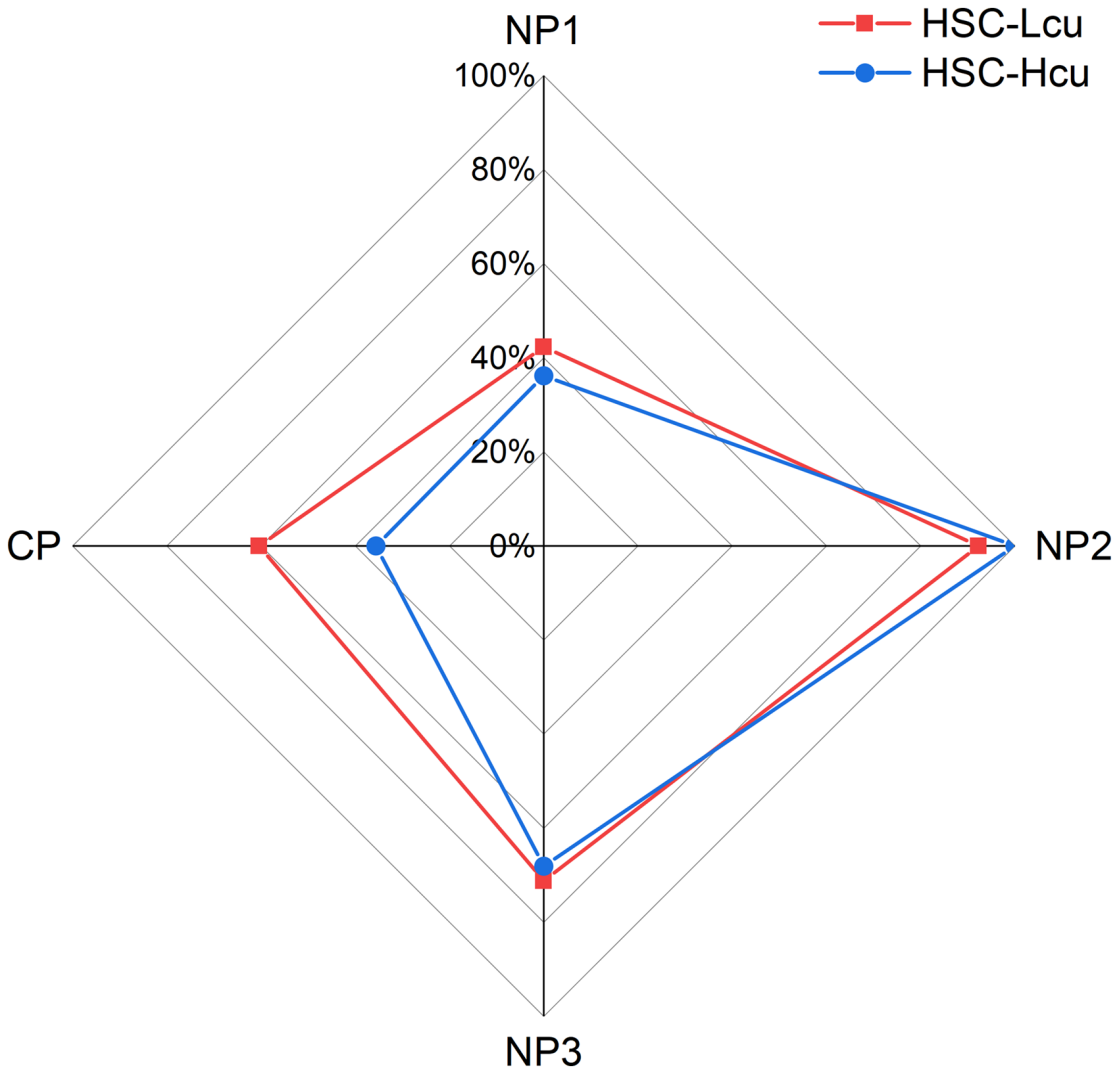
Proportion of null point and canal paresis presence in the HSC-Lcu group (red) and HSC-Hcu group (blue). CP, canal paresis; HSC-Lcu, horizontal semicircular canal light cupula; HSC-Hcu, horizontal semicircular canal heavy cupula; NP1, first null point; NP2, second null point; NP3, third null point.

In our study, 42.3% of patients with HSC-Lcu (*n* = 22) and 36.2% of patients with HSC-Hcu (*n* = 17) had the first null point (NP1). 93.2% of patients with HSC-Lcu (*n* = 48) and 100% of patients with HSC-Hcu (*n* = 47) had the second null point (NP2). 71.2% of patients with HSC-Lcu (*n* = 37) and 68.1% of patients with HSC-Hcu (*n* = 32) had the third null point (NP3). During the SRT, two patients with HSC-Lcu and three patients with HSC-Hcu demonstrated equal intensity of nystagmus on both the right and left sides. The side of stronger nystagmus intensity during the SRT was consistent with the side of the NP in 38 patients with HSC-Lcu and 21 patients with HSC-Hcu (Tables 2, 3; Figure 2). There was no significant difference in NP1, NP2, or NP3 between the HSC-Lcu and HSC-Hcu groups (Table 4).

**Table 2:** Nystagmus characteristics, null point, and caloric test of patients with HSC-Lcu.

NO.	Sex	Age	Strong side during the SRT	NP1	NP2	NP3	CP/D
1	F	84	L	+	L	L	ND
2	M	75	L	−	L	−	−
3	M	72	R	+	R	R	+/R
4	F	47	L	−	L	L	+/L
5	F	55	R	−	L	L	+/L
6	F	60	R	−	R	−	+/R
7	F	74	R	+	R	R	+/R
8	F	55	L	−	−	L	−
9	F	63	R	−	R	R	−
10	M	59	L	+	R	R	+/R
11	F	77	L	−	L	−	ND
12	F	56	R	−	R	R	+/R
13	F	75	R	+	R	R	+/R
14	F	76	R	−	R	−	+/R
15	F	73	L	+	L	L	+/L
16	F	76	L	−	L	L	−
17	F	65	R	+	R	R	+/R
18	F	62	L	−	L	−	+/L
19	F	48	L	−	L	−	ND
20	F	63	R	−	L	L	−
21	F	43	R	−	−	R	−
22	F	78	L	+	L	L	+/R
23	M	59	R	−	R	R	+/R
24	F	59	R	+	R	R	+/R
25	F	69	L	−	R	−	ND
26	M	81	L	+	L	L	−
27	M	73	R	−	L	L	+/R
28	F	62	L	+	R	−	−
29	M	58	R	+	R	R	−
30	M	67	R	−	R	R	+/R
31	F	74	L	−	L	−	+/L
32	M	67	−	+	R	R	−
33	M	59	R	+	L	−	+/L
34	M	60	L	−	L	L	+/L
35	F	55	L	−	−	L	−
36	F	51	R	+	R	R	−
37	M	76	L	−	R	−	+/L
38	F	73	R	−	L	−	+/L
39	F	71	R	+	R	R	−
40	F	62	L	−	L	−	+/R
41	F	70	R	−	L	L	+/L
42	F	69	R	+	R	R	−
43	M	98	R	−	−	R	+/R
44	F	76	L	+	L	L	−
45	F	68	L	+	R	R	+/R
46	F	64	R	+	L	L	−
47	F	67	R	+	R	R	−
48	M	74	L	−	L	L	−
49	M	51	−	−	L	−	+/L
50	F	69	L	−	L	L	−
51	M	74	L	+	L	L	+/L
52	M	72	R	−	R	−	+/R

−, no nystagmus observed on the supine position or the same nystagmus intensity on right and left roll tests or with canal paresis value < 25%. +, null point found or with canal paresis value ≥ 25%. CP, canal paresis; D, direction; HSC-Lcu, horizontal semicircular canal light cupula; F, female; L, left side; M, male; ND, not done; No., the serial number of patients; NP1, first null point; NP2, second null point; NP3, third null point; R, right side; SRT, supine roll test.

**Table 3:** Nystagmus characteristics, null point, and caloric test of patients with HSC-Hcu.

NO.	Sex	Age	Strong side during the SRT	NP1	NP2	NP3	CP/D
1	F	47	R	−	L	L	+/L
2	F	72	R	−	L	L	−
3	F	76	L	−	R	−	ND
4	M	74	L	−	L	−	−
5	F	62	R	−	R	R	−
6	M	70	R	−	R	R	−
7	M	78	L	−	R	R	+/R
8	F	76	L	−	R	R	+/L
9	M	68	L	+	L	L	+/R
10	M	64	R	−	L	L	−
11	F	72	L	−	L	L	+/L
12	M	90	−	+	R	R	−
13	M	59	L	−	R	R	−
14	F	71	L	+	R	R	+/L
15	M	71	R	+	R	R	+/L
16	M	42	R	−	L	−	+/L
17	F	72	L	−	R	R	−
18	F	67	L	−	R	−	+/R
19	F	66	L	−	L	L	−
20	M	83	R	−	L	−	−
21	M	69	L	+	L	L	+/R
22	M	71	−	−	L	L	−
23	M	70	L	−	L	−	+/L
24	F	51	L	+	L	L	−
25	F	62	R	−	L	−	−
26	F	54	R	+	L	L	−
27	F	68	L	+	L	L	−
28	M	72	R	−	R	R	−
39	F	62	L	−	L	L	−
30	F	70	L	−	R	−	−
31	F	73	R	+	L	L	−
32	F	74	R	+	L	L	−
33	M	59	L	+	L	L	−
34	M	53	−	+	R	R	+/R
35	F	61	R	−	R	R	−
36	M	76	L	+	R	R	−
37	M	81	L	−	L	−	+/R
38	M	73	R	+	R	−	+/L
39	M	80	R	+	L	L	+/R
40	M	72	L	−	R	−	−
41	F	63	L	−	L	L	ND
42	F	75	R	−	R	R	
43	M	47	R	−	L	−	+/L
44	F	70	R	+	L	−	−
45	M	82	R	+	R	R	−
46	M	71	L	−	R	−	−
47	F	59	R	−	R	−	−

−, no nystagmus observed on the supine position or the same nystagmus intensity on right and left roll tests or with canal paresis value < 25%. +, null point found or with canal paresis value ≥ 25%. CP, canal paresis; D, direction; HSC-Lcu, horizontal semicircular canal light cupula; F, female; L, left side; M, male; ND, not done; No., the serial number of patients; NP1, first null point; NP2, second null point; NP3, third null point; R, right side; SRT, supine roll test.

**Table 4:** Comparison of the presence proportion of null point, and canal paresis between patients with HSC-Lcu and HSC-Hcu.

NP and CP	HSC-Lcu	HSC-Hcu	*p* value
NP1, *n* (%)	22 (42.3)	17 (36.2)	0.533
NP2, *n* (%)	48 (92.3)	47 (100)	0.052
NP3, *n* (%)	37 (71.2)	32 (68.1)	0.740
CPᵃ, *n* (%)	29 (65.9)	16 (37.2)	0.030^*^

^ᵃ^In the caloric test, we finally included 44 patients with HSC-Lcu and 43 patients with HSC-Hcu. ^*^p < 0.05. CP, canal paresis; HSC-Lcu, horizontal semicircular canal light cupula; HSC-Hcu, horizontal semicircular canal heavy cupula; n, number; NP1, the first null point; NP2, the second null point; NP3, the third null point.

### 3.4 Caloric test findings

Forty-eight patients with HSC-Lcu were eligible for caloric testing; of these, four were excluded due to intolerance (*n* = 3) or cerumen impaction (*n* = 1), leaving 44 patients included in the analysis. Among them, 65.9% (29/44) had CP, and in 56.8% (25/44), the CP side was ipsilateral to the affected side (Table 2). For the HSC-Hcu group, 45 patients were initially eligible for the caloric test. Two patients were excluded due to intolerance, resulting in 43 patients analyzed. Among them, 37.2% (16/43) had CP, and in 18.6% (8/43), the CP side was ipsilateral to the affected side (Table 3). CP was more frequently observed in patients with HSC-Lcu than in those with HSC-Hcu (*p* = 0.030) (Table 4).

### 3.5 Multivariable regression analysis

Multivariable logistic regression analysis assessed factors associated with subtype classification. The Hosmer-Lemeshow test confirmed adequate model fit (χ^2^ = 10.729, df = 8, *p* = 0.218). The stroke history revealed a non-significant positive correlation with the HSC-Lcu classification (OR = 3.930, 95% CI: 0.405–38.111, *p* = 0.238), with five cases observed in the HSC-Hcu group compared to one case in the HSC-Lcu group. Demographic factors were not significantly associated with subtype differentiation. Age: OR = 0.979 (95% CI: 0.932–1.029, *p* = 0.410); male sex: OR = 1.874 (95% CI: 0.741–4.738, *p* = 0.185). The NP1 and NP 3 factors were also not significantly associated with subtype differentiation. NP1: OR = 0.626 (95% CI: 0.235–1.669, *p* = 0.626); NP3: OR = 0.992 (95% CI: 0.338–2.916, *p* = 0.989). Complete separation occurred for SSNHL (exclusively in HSC-Lcu group) (OR = 0, 95% CI: 0–, *p* > 0.999) and NP2 (all present in HSC-Hcu group) (OR = 1.56 × 10^9^, 95% CI: 0–, *p* > 0.999), resulting in non-interpretable estimates (Table 5).

**Table 5:** Multivariable logistic regression model for HSC-cupula subtype classification.

Variable	β	OR [95% CI]	*p* value
Age, years	−0.21	0.979 [0.932–1.029]	0.410
Sex (male)	0.628	1.874 [0.741–4.738]	0.185
Presence of SSNHL history	−21.004	0 [0-]	>0.999^a^
Presence of stroke history	1.369	3.930 [0.405–38.111]	0.238
Presence of NP1	−0.468	0.626 [0.235–1.669]	0.349
Presence of NP2	21.173	1.56 × 10^9^ [0-]	>0.999^a^
Presence of NP3	−0.008	0.992 [0.338–2.916]	0.989
CP value, percentage	0.010	1.010 [0.987–1.034]	0.384

Reference group: HSC-Hcu (coded as 0). ^a^Complete separation occurred for SSNHL (exclusively in HSC-Lcu group) and NP2 (all present in HSC-Hcu group), resulting in non-interpretable estimates. β, regression coefficient; CI, confidence interval; HSC, horizontal semicircular canal; Lcu, light cupula; Hcu, heavy cupula; SSNHL, sudden sensorineural hearing loss; OR, odds ratio; NP, null point; CP, canal paresis.

## 4 Discussion

We explored the clinical characteristics and compared CP using caloric tests in patients with HSC-Lcu and HSC-Hcu. Our key findings are: (1) no significant differences in the history of most peripheral vestibular disorders (BPPV, vestibular neuritis, Meniere’s disease, otitis media) or vestibular migraine between HSC-Lcu and HSC-Hcu groups; (2) a significantly higher prevalence of SSNHL history in HSC-Lcu compared to HSC-Hcu; (3) no significant difference in the presence or type of between groups; (4) the affected side of cupula is unreliable when determined by the nystagmus intensity in the SRT; and (5) a higher frequency of CP on caloric testing in HSC-Lcu, often ipsilateral to the affected side.

In our study, the history of peripheral vestibular disorders and otologic disease was present in 18 (34.6%) patients with HSC-Lcu, including SSNHL (*n* = 10, 19.2%), BPPV (*n* = 1, 1.9%), vestibular neuritis (*n* = 5, 9.6%), Meniere’s disease (*n* = 2, 3.8%), otitis media (*n* = 2, 3.8%) and in 13 (27.7%) patients with HSC-Hcu, including BPPV (*n* = 8, 8.5%), vestibular neuritis (*n* = 3, 6.4%), Meniere’s disease (*n* = 1, 2.1%), otitis media (*n* = 1, 2.1%). Si (10) included 30 patients with persistent geotropic DCPN and 44 patients with persistent apogeotropic DCPN, reporting that the history of peripheral vestibular disorders and otologic disease was present in 18 (40.9%) patients with persistent apogeotropic-DCPN (BPPV, *n* = 15, 34.1%; vestibular neuritis, *n* = 3, 6.8%) and 4 (13.3%) patients with persistent geotropic DCPN (BPPV, *n* = 2, 6.7%; vestibular neuritis, *n* = 1, 3.3%). Peng (24) included 189 participants with persistent geotropic DCPN, reporting 120 (63.5%) participants with positional vertigo history. Kim (25) included 13 patients with acute otitis media, reporting 1 (7.7%) patients with persistent geotropic DCPN and 2 (15.4%) patients with persistent apogeotropic DCPN. These results suggest that the history of peripheral vestibular disorders and otologic disease was common in patients with persistent DCPN.

Tomanovic (26) followed up 20 subjects who suffered persistent DCPN and found that eight (40%) of the 20 patients had a history of vestibular migraine (VM). Radtke (7) found positional nystagmus in 28% of VM patients, and they believed that the underlying changes might be the vasospasms of the inner ear and/or the brainstem. In our study, the history of VM was also present in patients with HSC-Lcu and HSC-Hcu, indicating that VM was related to a dysfunctional peripheral vestibule.

We found 1 (1.9%) patients with HSC-Lcu and 5 (10.6%) patients with HSC-Hcu with a history of stroke. The study of Si (10) reported that a history of diseases associated with atherosclerosis was present in 17 (38.6%) patients with persistent apogeotropic DCPN and 4 (13.3%) patients with persistent geotropic DCPN. The mean age of the study participants we included was 66.6 years of HSC-Lcu and 68.0 years of HSC-Hcu, and age is a risk factor for cerebrovascular disease. The participants we recruited were from the neurology department, which may also caused a high rate of history of stroke in participants. In our multivariable logistic regression analysis, the stroke history revealed a non-significant positive correlation with the HSC-cupula classification (OR = 3.930, 95% CI: 0.405–38.111, *p* = 0.238). The relationship between cupula and stroke is unclear and needs to be explored in future studies.

The history of SSNHL was more frequent in the HSC-Lcu group (*n* = 8) than in the HSC-Hcu group (*p* = 0.005). It highlights a potential pathophysiological link between the two conditions. SSNHL is characterized by acute cochlear damage, which is often attributed to viral infections, ischemia, hemorrhage, or disruption to the blood-labyrinth barrier (27). Importantly, our findings are consistent with previous reports of SSNHL occurring alongside persistent geotropic DCPN (8, 9, 28). Böhmer (28) reported three patients with complete sudden unilateral hearing loss who presented with geotropic positional nystagmus. A retrospective study by Chang-Hee Kim (9) identified 17 patients with ipsilateral SSNHL and geotropic DCPN between 2006 and 2013.

The consistent association of SSNHL with the light cupula indicates the presence of shared mechanisms. Kim (29) proposed that SSNHL may increase endolymphatic specific gravity via plasma protein leakage into the endolymph. This is supported by 3D-FLAIR MRI studies that detect high signals indicating the presence of proteins or blood products in the inner ear of patients with SSNHL (30, 31), which is likely due to a breakdown in the blood-labyrinth barrier (32). An elevated endolymphatic specific gravity would make the cupula relatively “lighter,” which would explain persistent geotropic DCPN (29, 33). Yong Won Kim reported a patient with left-sided SSNHL and persistent geotropic DCPN observed during the SRT. Three weeks after SSNHL onset, the SRT revealed persistent apogeotropic DCPN. The author suggested that this transformation may result from an initial increase in endolymphatic specific gravity, followed by overcompensation of endolymphatic homeostasis. This overcompensation could lead to a specific gravity lower than that of the cupula, thereby resulting in apogeotropic DCPN (8). Heavy cupula usually develops as a result of direct cupular densification (e.g., cupulolithiasis) (33), which is not related to endolymph changes associated with SSNHL. This distinction explains why there was no history of SSNHL in our HSC-heavy cupula group. These results improve the clinical understanding etiologies of HSC-Lcu and HSC-Hcu. The strong association between SSNHL history and HSC-Lcu necessitates careful otological evaluation, including audiometry, in patients presenting with persistent geotropic DCPN. Conversely, identifying HSC-Lcu should prompt inquiry about recent hearing changes. Future studies should include longitudinal research involving serial audiometry and vestibular testing in SSNHL patients to elucidate the natural history of associated cupulopathies. Advanced imaging studies using 3D-FLAIR MRI could correlate inner ear signal abnormalities with specific gravity changes and cupula type. The exploration of biomarkers of blood-labyrinth barrier integrity in HSC-Lcu patients with and without SSNHL is also required.

Light or heavy cupula, and their corresponding affected sides, can be diagnosed by identifying the NP. However, NP1 does not assist in determining the affected side (13, 17, 34). When the head is slightly turned toward the affected side such that the cupula of the horizontal semicircular canal aligns with the gravitational vector, the nystagmus ceases—this position is referred to as the null point (NP2 or NP3) (35). Our study found no significant difference in the presence of NP1, NP2, or NP3 between the HSC-Lcu and HSC-Hcu groups, which is consistent with previous study (10). This may be because, regardless of whether NP1, NP2, or NP3 is observed, each reflects a position where the specific gravity of the cupula reaches equilibrium with that of the endolymph—regardless of whether the endolymph has a higher or lower specific gravity than the cupula itself (the underlying mechanism of the light or heavy cupula).

According to previous studies, the NP side is ipsilateral to the affected side in both light and heavy cupula cases (12–16). Some studies have also proposed determining the affected side of persistent geotropic or apogeotropic DCPN based on nystagmus intensity during the SRT: in light cupula, the affected side is considered ipsilateral to the side with stronger nystagmus intensity, whereas in heavy cupula, it is considered contralateral (17, 36). However, in our study, two patients with HSC-Lcu and three patients with HSC-Hcu showed equal nystagmus intensity on both the right and left sides. The side of stronger nystagmus intensity during the SRT was consistent with the affected side in 73.1% of patients with HSC-Lcu (*n* = 38) and 44.7% of patients with HSC-Hcu (*n* = 21). In our previous study, the accuracy rate of lateralization through the sides with more vigorous DCPN in the SRT was 63.5%, significantly lower than through NP (*p* < 0.001) (37). Other studies have also found that determining the affected side through the SRT is unreliable (14, 17, 38). According to Ewald’s second law, angular acceleration that causes an utriculofugal deviation of the cupula evokes more intense nystagmus than utriculopetal deviation (39). A previous study demonstrated that patients with total unilateral canal paresis exhibit utriculofugal–utriculopetal asymmetry only when the nystagmus reaches at least 60°/s in SPV during horizontal head accelerations (39). The contradictory results observed in our study may be due to the lower magnitude of cupula deviation, which may not be sufficient to produce a corresponding utriculofugal–utriculopetal asymmetry.

CP was assessed to evaluate peripheral vestibular function (22). In the caloric test, we included 44 patients with HSC-Lcu and 43 patients with HSC-Hcu. We found that 65.9% of patients (*n* = 29) with HSC-Lcu had CP, and in 56.8% of these patients (*n* = 25), the CP side was ipsilateral to the affected side. In comparison, 37.2% of patients (*n* = 16) with HSC-Hcu had CP, and in 18.6% of these patients (*n* = 8), the CP side was ipsilateral to the affected side. Tomanovic (26) studied 20 patients with persistent geotropic DCPN and found that 65% (*n* = 13) had CP. In caloric testing conducted by Ichijo H., 21% of 14 patients with HSC-Lcu showed CP on the side opposite the lesion, while no patients with HSC-Hcu had CP (22). In another study, Si (10) included 30 patients with persistent geotropic DCPN and 44 patients with persistent apogeotropic DCPN, reporting that 43.3% (*n* = 13) of those with persistent geotropic DCPN and 15.9% (*n* = 7) of those with persistent apogeotropic DCPN had CP. These findings also showed a higher proportion of CP in light cupula than in heavy cupula. In our study, CP was more frequently observed in patients with HSC-Lcu than in those with HSC-Hcu (*p* = 0.030), which is consistent with previous findings. However, when evaluated within a multivariable logistic regression model designed to identify distinct predictors of the HSC-cupula subtype, the presence of CP was not found to be statistically significantly associated with the outcome (*p* > 0.05). This discrepancy probably arises because the regression model adjusts for other variables. Crucially, the model included near-perfect predictors: SNHL history was exclusive to the HSC-Lcu group, while NP2 was present in all of the HSC-Hcu group. These dominant predictors strongly influence subtype differentiation within the model, potentially obscuring the independent contribution of CP and making it difficult to estimate its effect size accurately. While the univariate association indicates that CP is linked to the HSC-Lcu subtype, its utility for independent prediction diminishes when considered alongside these highly specific features (SSNHL history for HSC-Lcu and NP2 for HSC-Hcu). The univariate association remains clinically relevant, indicating a link between the subtype and peripheral vestibular function as measured by caloric testing. We suggest that the mechanism of light cupula may be more closely related to cupula dysfunction compared to heavy cupula.

## 5 Conclusion

A history of SSNHL is significantly associated with HSC-Lcu, suggesting shared pathophysiology distinct from HSC-Hcu. A bigger sample size study is still needed to confirm this result. The affected side of cupula can be determined by the nystagmus intensity during the SRT is unreliable. CP occurs more frequently and often in HSC-Lcu, indicating that the function of HSC-Lcu was more impaired than that of HSC-Hcu. Clinicians should consider SSNHL history and perform caloric testing when evaluating suspected HSC-Lcu, and rely on the NP for lateralization.

### 5.1 Study limitations

This study has several limitations. Reliance on medical records for historical data (e.g., sudden SSNHL or vestibular migraine) introduces the potential for recall or documentation bias. A definitive diagnosis of SSNHL requires strict audiometric criteria to be met within 72 h, and these criteria may not have been uniformly documented retrospectively. The study of the association of ISSNHL and CP was worthwhile, but the small amount of data we have on ISSNHL does not allow us to draw reliable results and conclusions. Future studies could include patients with light cupula combined with ISSNHL, study the interrelationship between ISSNHL and CP, and provide a reference for the pathogenesis of light cupula combined with ISSNHL. The sample size of the multivariable logistic regression model is inadequate. We included eight predictors, so the sample size should be at least 80 per group. Also, the small sample size resulted in no patients with a history of SSNHL in the HSC-Hcu group, and NP2 was present in all HSC-Hcu patients. Resulting in non-interpretable estimates in the multivariable logistic regression analysis. Larger sample sizes should be included in future studies.

## Data Availability

The original contributions presented in the study are included in the article/supplementary material, further inquiries can be directed to the corresponding authors.
